# Coagulopathy in Zellweger spectrum disorders: a role for vitamin K

**DOI:** 10.1007/s10545-017-0113-8

**Published:** 2017-11-14

**Authors:** Sara Zeynelabidin, Femke C. C. Klouwer, Joost C. M. Meijers, Monique H. Suijker, Marc Engelen, Bwee Tien Poll-The, C. Heleen van Ommen

**Affiliations:** 1 Department of Pediatric Neurology, Emma Children’s Hospital, Academic Medical Center, University of Amsterdam, Meibergdreef 9, 1105 AZ Amsterdam, The Netherlands; 2 Department of Pediatric Hematology, Emma Children’s Hospital, Academic Medical Center, Amsterdam, The Netherlands; 3 Laboratory Genetic Metabolic Diseases, Academic Medical Center, University of Amsterdam, Meibergdreef 9, 1105 AZ Amsterdam, The Netherlands; 4 Department of Experimental Vascular Medicine, Academic Medical Center, University of Amsterdam, Meibergdreef 9, 1105 AZ Amsterdam, The Netherlands; 5 Department of Plasma Proteins, Sanquin Research, Amsterdam, the Netherlands; 6 Department of Pediatric Hematology, Erasmus MC Sophia Children’s Hospital, Rotterdam, The Netherlands

**Keywords:** Zellweger spectrum disorders, Coagulopathy, Vitamin K, Peroxisome biogenesis disorders

## Abstract

**Introduction:**

Zellweger spectrum disorders (ZSDs) are caused by an impairment of peroxisome biogenesis, resulting in multiple metabolic abnormalities. This leads to a range of symptoms, including hepatic dysfunction and coagulopathy. This study evaluated the incidence and severity of coagulopathy and the effect of vitamin K supplementation orally and IV in ZSD.

**Methods:**

Data were retrospectively retrieved from the medical records of 30 ZSD patients to study coagulopathy and the effect of vitamin K orally on proteins induced by vitamin K absence (PIVKA-II) levels. Five patients from the cohort with a prolonged prothrombin time, low factor VII, and elevated PIVKA-II levels received 10 mg of vitamin K IV. Laboratory results, including thrombin generation, at baseline and 72 h after vitamin K administration were examined.

**Results:**

In the retrospective cohort, four patients (13.3%) experienced intracranial bleedings and 14 (46.7%) reported minor bleeding. No thrombotic events occurred. PIVKA-II levels decreased 38% after start of vitamin K therapy orally. In the five patients with a coagulopathy, despite treatment with oral administration of vitamin K, vitamin K IV caused an additional decrease (23%) of PIVKA-II levels and increased thrombin generation.

**Conclusion:**

Bleeding complications frequently occur in ZSD patients due to liver disease and vitamin K deficiency. Vitamin K deficiency is partly corrected by vitamin K supplementation orally, and vitamin K administered IV additionally improves vitamin K status, as shown by further decrease of PIVKA-II and improved thrombin generation.

## Introduction

Zellweger spectrum disorders (ZSD) are a group of autosomal recessively inherited disorders with a deficiency of functional peroxisomes caused by mutations in one of the peroxisome assembly protein (*PEX*) genes. (Wanders and Waterham 2006). One of the biochemical consequences of peroxisomal dysfunction is accumulation of very long-chain fatty acids and bile acid intermediates [e.g., dihydroxycholestanoic acid (DHCA) and trihydroxycholestanoic acid (THCA)], contributing to a variety of symptoms with a spectrum of severity, including liver disease, developmental delay, and hearing and vision deficits (Klouwer et al. 2015).

In general, severe liver disease is associated with impairment of coagulation, since the liver synthesizes multiple coagulation factors. Deficiencies of fat-soluble vitamins are common among ZSD patients (Klouwer et al. 2015), and vitamin K deficiency may coexist and partially attribute to the coagulopathy in these patients. Most ZSD patients therefore receive supplementation orally of vitamin K and other fat-soluble vitamins (Berendse et al. 2016). Although ZSD patients most likely have an increased bleeding diathesis, recent studies state coagulation rebalance occurs in patients with liver disease, with higher risks of both bleeding and thrombosis (Lisman and Porte 2011; Magnusson et al. 2016). The presence of hemorrhages, thrombosis, or effect of vitamin K supplementation orally have not been systematically studied in ZSD patients. Intravenous (IV) administration of vitamin K might be more effective than orally, as shown by Pereira et al. in adults with liver cirrhosis (Pereira et al. 2003). In this study, subclinical vitamin K deficiency was corrected in 15 of 16 patients receiving 10 mg vitamin K IV compared with three of 15 receiving oral supplementation. In another study, infants with hyperbilirubinemia had lower vitamin K concentrations after oral versus IV administration of vitamin K, reflecting the low efficiency of intestinal absorption (Pereira et al. 2005).

We retrospectively evaluated the incidence and severity of bleeding and thrombotic complications, the pathogenesis of coagulopathy, and the effect of vitamin K supplementation orally in ZSD patients. Furthermore, the effect of vitamin K IV was prospectively studied in five ZSD patients.

## Patients and methods

### Study design

We retrospectively investigated the incidence and severity of bleeding and thrombotic complications in a cohort of ZSD patients at the Academic Medical Center (AMC) in Amsterdam, The Netherlands. Furthermore, we studied the pathogenesis of coagulopathy in these patients. We hypothesized that the levels of DHCA and THCA are correlated with liver disease severity in these patients and consequently to prothrombin time (PT) and other liver parameters. In addition, the effect of vitamin K supplementation orally on coagulation parameters was evaluated. We then prospectively studied the effect of vitamin K IV on coagulation parameters and thrombin generation in five ZSD patients. The Medical Ethics Committee of the AMC approved this pilot study. Written informed consent was obtained from all parents of enrolled patients.

### Methods

#### Retrospective study

The Academic Medical Center in Amsterdam is the expertise center in The Netherlands for peroxisomal disorders. An expanding cohort of ZSD patients is followed here and are seen at the outpatient clinic at least twice a year. Patient data were retrospectively collected by reviewing the medical records of 30 ZSD patients. All patients with enough data available for analyses were included, and all received vitamin K supplementation orally (mixtura phytomenadioni 10 mg/ml base arachis oil). The starting dose was 1 mg per week and titrated based on symptoms and laboratory tests, including PT and/or proteins induced by vitamin K absence (PIVKA-II) levels. Data included age, gender, *PEX* gene mutation, type and severity of bleeding and thrombotic complications, vitamin K supplementation dose, and the following laboratory parameters: PT, factor V (FV), factor VII (FVII), aspartate aminotransferase (AST), alanine aminotransferase (ALT), DHCA and THCA concentrations, and PIVKA-II levels before and after initiating vitamin K therapy orally. Bleeding was categorized as major, clinically relevant, nonmajor, or minor bleeding according to the Perinatal and Pediatric Subcommittee of the Scientific and Standardization Committee of the International Society on Thrombosis and Hemostasis criteria (Mitchell and Male 2011).

#### Prospective pilot study

For this proof of principle pilot study, five patients with ZSD aged ≥5 years with a prolonged PT, low FVII, and elevated PIVKA-II levels were included for the prospective pilot study. All patients received 10 mg IV in 10 min of phytonadione (vitamin K_1_, Konakion MM) dissolved in 0.9% sodium chloride. Blood samples were obtained by venepuncture before and 72 h after administration. Blood was collected in plastic tubes containing trisodium citrate or ethylenediaminetetraacetate (EDTA). Coagulation studies included evaluation for PT, activated partial thromboplastin time (APTT), FV, FVII, factor IX (FIX), factor X (FX), PIVKA-II, platelet count, D-dimer, fibrinogen, and calibrated automated thrombography (CAT).

### Coagulation assays

Platelet count and coagulation analyses including PT, APTT, FV, FVII, FIX, FX, d-dimer, and fibrinogen were determined in the central laboratory of the hospital. In the retrospective study, PIVKA-II levels were determined by enzyme-linked immunosorbent assay (ELISA) (Asserachrom PIVKA-II, Diagnostica Stago, France). As these kits were discontinued, in the prospective study, PIVKA-II levels were determined by ELISA with a kit from Kamiya Biomedical Company (PIVKA-II, Japan). In vitro thrombin generation was determined with CAT, as described (Hemker et al. 2003). Coagulation was triggered by recalcification in the presence of 1 or 5 pM recombinant human tissue factor (Innovin, Siemens Healthcare Diagnostics, Marburg, Germany), 4 μM phospholipids, and 417 μM fluorogenic substrate Z-Gly-Gly-Arg-AMC (Bachem, Bubendorf, Switzerland). Fluorescence was monitored using a Fluoroskan Ascent fluorometer (ThermoLabsystems, Helsinki, Finland), and thrombin generation parameters (lag time, peak height, endogenous thrombin potential, velocity index) were calculated using Thrombinoscope software (Maastricht, The Netherlands).

### Statistical analysis

In the retrospective study, demographic data were analyzed using descriptive statistics. Spearman’s correlation coefficients were calculated to assess the strength of relations between last measured DHCA or THCA and severity of liver dysfunction (PT, AST, ALT, FV, FVII). In both studies, Wilcoxon signed-rank test was used to compare samples before and after therapy. *P* ≤ 0.05 was considered statistically significant. Statistical analysis was performed with SPSS (IBM SPSS statistics version 22). All collected data are displayed as graphs using levels before and after vitamin K therapy [GraphPad Prism (Prism 5 for Mac OS X)].

## Results

### Retrospective study

Thirty ZSD patients were assessed in the retrospective study. Mean age was 14.0 ± 8.8 years. Most patients had mutations in *PEX1* (*n* = 24); patient characteristics are shown in Table 1.

**Table 1:** Patient characteristics

Variable	Reference values	Results [mean (median; min, max) ± SD]	Patients with abnormal value/total (%)
Age in years, *n* = 30		14.0 (14; 0, 33) ± 8.8	
Gender		Male, 15	
		Female, 15	
*PEX* gene, * n* patients		*PEX1*, 24	
		*PEX6*, 1	
		*PEX10*, 1	
		*PEX11*, 1	
		*PEX26*, 1	
PIVKA-II in μg/L, before vit K orally	<2.5	11.3 (6.6; 2.5, 34.6) ± 10.6	17/17 (100%)
PIVKA-II in μg/L, after vit K orally	<2.5	4.1 (2.7; 1.0, 17.4) ± 4.0	11/20 (55%)
PT, in s	9.7–11.6	12.5 (11.9; 10.8, 19.3) ± 1.8	23/30 (77%)
AST in U/L	0–40	52 (46; 0, 178) ± 33.5	17/29 (59%)
ALT in U/L	0–45	41 (32; 16, 125) ± 27.8	9/30 (30%)
Factor V in %	80–140	81 (85; 24, 134) ± 25.5	11/30 (37%)
Factor VII in %	80–140	71 (78; 5, 110) ± 28.9	17/30 (57%)
DHCA in μmol/L	0.0–0.0	2.2 (0.6; 0.0, 17.5) ± 4.0	25/30 (83%)
THCA in μmol/L	0.0–0.1	4.0 (0.3; 0.0, 37.4) ± 8.4	28/30 (93%)
Minor bleeding			14/30 (47%)
Major bleeding			4/30 (13%)

*SD* standard deviation, *PEX* peroxisome assembly protein,* PIVKA-II* proteins induced by vitamin K absence,* PT* prothrombin time,* AST* aspartate aminotransferase,* ALT* alanine aminotransferase, * DHCA* dihydroxycholestanoic acid,* THCA* trihydroxycholestanoic acid,* SD* standard deviation

#### Bleeding and thrombotic complications

Four of the 30 patients (13.3%) developed an intracranial hemorrhage, of which three were spontaneous. Characteristics and laboratory parameters are shown in Table 2. Patient 1 died at the age of 3 months as result of an intracerebral hemorrhage in the right frontal lobe with cerebral herniation. PIVKA-II levels were elevated, and PT was prolonged. Patient 2 recovered from a small subdural hemorrhage at the age of 6 years, with elevated PIVKA-II levels and normal PT and platelets levels. Patient 3 had a traumatic skull fracture with two epidural hemorrhages. Lab results showed decreased number of platelets, increased PT, low FV, but normal PIVKA-II. After a few hours, the hemorrhages enlarged with signs of herniation, leading to a surgical decompression procedure. Despite vitamin K therapy orally and extra vitamin K IV beforehand, bleeding during surgery was hard to stop. She was treated with tranexamic acid and received several platelet, erythrocyte, and fresh–frozen plasma transfusions and recovered after surgery. This patient had normal PIVKA-II levels in prior years. Patient 4, born after 34 weeks and 2 days, developed grade 1 intraventricular bleeding at the age of 4 days, which increased to grade 3 1 day later. Conservative therapy led to improvement; no laboratory results were available for this patient at the time of the bleeding.

**Table 2:** Clinical and laboratory parameters in Zellweger spectrum disorders (ZSD) patients with major bleeding complications

	Reference values	Patient 1	Patient 2	Patient 3	Patient 4
Age at intracranial bleeding		3 months	6 years	7 years	4 days, premature at 34 weeks
Gender		Male	Female	Female	Male
Cause of bleeding		Spontaneous	Spontaneous	Traumatic	Spontaneous
Type of bleeding		Intracerebral hemorrhage with cerebral herniation	Small subdural hemorrhage	Two epidural hematomas	Intraventricular hemorrhage grade 3
Outcome		Died	Recovered	Surgery, recovered	Recovered
Vitamin K supplementation		7 mg/week	7 mg/week	70 mg/week	–
PT	9.7–11.6 s	20.2	13.9	16.2	–
PIVKA-II	<2.5 μg/L	>7360	181	1.7*	–
FV	80–140%	114	51	34**	–
FVII	80–140%	8	26	35**	–
Platelet count	150–450 × 10^9^/L	573	218	91	–

*PT* prothrombin time,* PIVKA-II* proteins induced by vitamin K absence,* FV* factor V,* FVII * factor VII

*Determined 3 years before the bleeding

**Determined 1 year before the bleeding

Minor bleeding complications, such as nosebleeds, bruises, and gingiva bleedings, occurred in 14 patients (46.7%). None of the 30 ZSD patients developed thrombotic events.

#### Correlation between bile acid intermediates and liver parameters

**Fig. 1:**
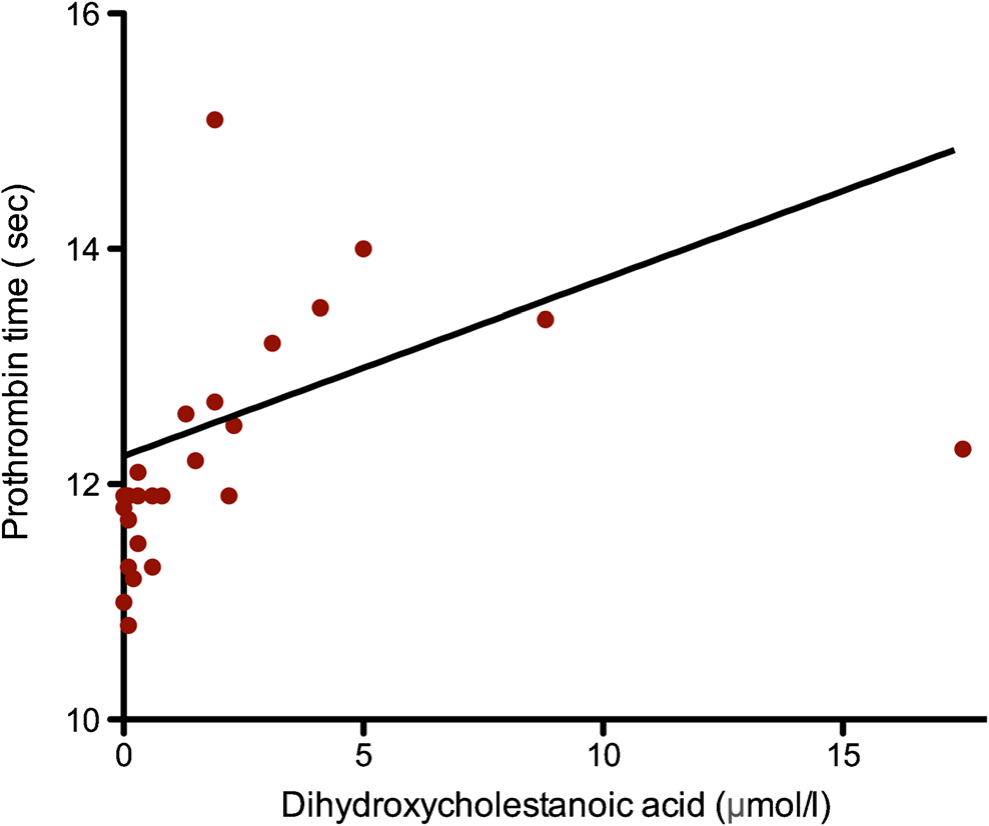
Relationship between last measured dihydroxycholestanoic acid (DHCA) (*x* axis, normal range 0.00–0.12 μmol/L) and prothrombin time (PT) (*y* axis, normal range 9.7–11.6 s).* Trend line* indicates a significant correlation of DHCA and PT (*r* = 0.76, *p* < 0.001)

Plasma levels of DHCA (*r* = 0.76, *p* < 0.001, Fig. 1) and THCA (*r* = 0.79,* p* < 0.001) are significantly correlated with PT. AST (*r* = 0.49, *p* = 0.004), ALT (*r* = 0.47, *p* = 0.005), FV (*r* = −0.45, *p* = 0.006), and FVII (*r* = −0.77,* p* < 0.001) also show significant correlations with DHCA. Similar correlations were seen with THCA (data not shown).

#### Effect of orally administered vitamin K therapy

**Fig. 2:**
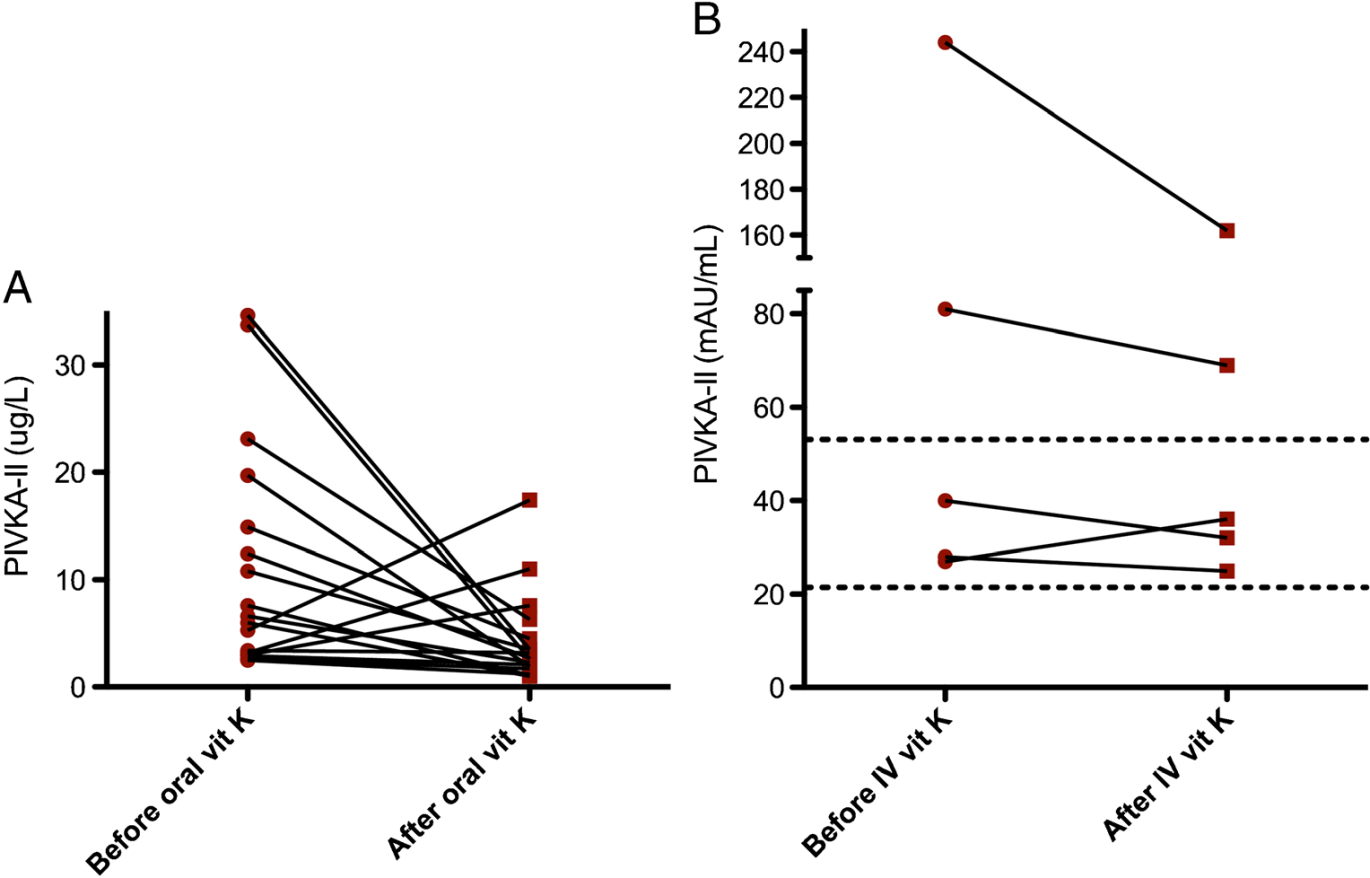
**a** Proteins induced by vitamin K absence (PIVKA-II) of 17 patients before (*dots*) and after (*squares*) vitamin K therapy administered orally. Cutoff range is <2.5 μg/L. Almost all patients showed a decreased PIVKA-II level (*p* ≤ 0.05). **b** PIVKA-II levels before (*T* = 0) and after (*T* = 72 h) vitamin K supplementation IV.* Interrupted lines* indicate reference range of 21–56 mAU/ml

PIVKA-II levels were measured before and after initiation of vitamin K orally in 17 patients. The time interval between measurements varied from 6 to 10 years. Before therapy, all patients had increased levels of PIVKA-II (Fig. 2a). Median PIVKA-II level decreased by 38% after start of therapy: to normal levels in seven patients and to slightly above normal levels in seven; in three patients (20%), they increased. There was no significant correlation between the PIVKA-II level and the vitamin K dose (*r* = −0.379, *p* = 0.091) based on data from 14/17 patients).

### Prospective pilot study

#### Effect of intravenously administered vitamin K therapy

**Fig. 3:**
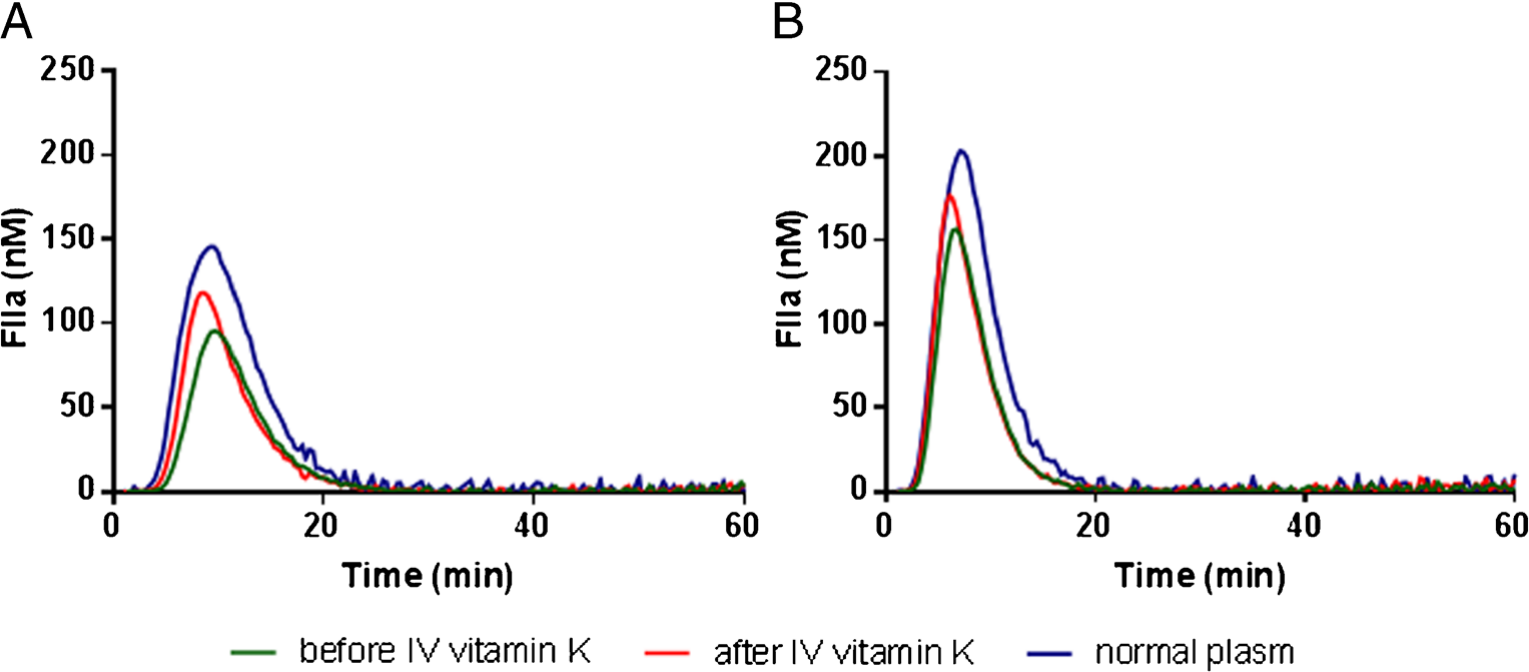
Thrombin generation analysis of Zellweger spectrum disorders (ZSD) patients. Thrombin generation was initiated with **a**1 pM or** b** 5 pM tissue factor. Thrombin generation was averaged for five patients before and after 72 h of vitamin K therapy IV

Five children (male:female = 1:4), mean age 7.6 years [standard deviation (SD 1.7)] received vitamin K IV. Four patients had *PEX1* mutations and one *PEX10* mutation. The oral dose varied between 1 mg administered twice a week and 13 mg/day. There is a significant correlation between vitamin K dose orally and PIVKA-II levels in these patients on *t* = 0 before IV vitamin K therapy (*r* = −0.900, *p* = 0.019). No differences were seen in global coagulation parameters before and 72 h after vitamin K. Mean PIVKA-II levels decreased 23%, from 84 to 64.8 mAU/ml (Fig. 2b). After vitamin K IV, thrombin generation improved, with earlier onset (lag time *p* = 0.223), faster rate of thrombin generation (velocity index *p* = 0.043) reaching higher peak thrombin values (peak* p* = 0.043), and a higher total amount of thrombin formed (endogenous thrombin potential* p* = 0.043) (Fig. 3).

## Discussion

This is the first study to investigate the incidence and severity of bleeding and thrombotic complications in ZSD patients. Major intracranial bleedings developed in 13.3%, including one patient born prematurely as a confounding risk factor, and minor bleedings in almost 50% of patients with a ZSD. These are most likely underreported due to the study’s retrospective design. None of the 30 ZSD patients developed thrombotic events. However, most patients were young, and their risk of late-onset thrombosis is unknown. The coagulopathy is partly caused by liver disease, probably due to accumulation of toxic bile-acid intermediates (Ferdinandusse et al. 2009). PT was prolonged in most patients and more prolonged in patients with higher bile-acid intermediates. Although PT can be used to investigate a loss of function in the procoagulant system, it does not predict the risk of bleeding in patients with liver disease (Lisman and Porte 2011). It is known that children with liver disease can develop severe bleeding as well as thrombotic complications (Magnusson et al. 2016). Reduced synthetic capability of liver cells results in decreased levels of both procoagulant and anticoagulant factors. In addition, hyperfibrinolysis may occur in children with chronic liver disease, especially if the disease is cirrhotic (El-Sayed et al. 2013). Whether a pediatric patient with liver disease and loss of reserve capacity develops bleeding or thrombosis depends on the vascular bed and additional risk factors in the individual patient (Magnusson et al. 2016). In children, various diseases are associated with liver failure, including infections, cholestatic disorders, metabolic disorders, and ischemia due to cardiac diseases. These diseases are different than those seen in adults and vary with age. In some diseases, bleedings occur more often, for example, in biliary atresia or α1-antitrypsin deficiency (Fischler and Lamireau 2014; Magnusson et al. 2016). Other diseases, such as cystic fibrosis, are more frequently linked to thrombotic events (Takemoto 2012). Many metabolic disorders are associated with bleeding complications (Croffie et al. 1999; Preston et al. 2013), and our study extended those findings by showing that in ZSD patients, bleeding complications are more prevalent than thromboses.

Impaired hepatic synthesis is not the only cause of coagulopathy in ZSD patients. The many risk factors for developing vitamin K deficiency, including steatorrhea, poor oral intake of the vitamin and frequent antibiotic treatment. PIVKA-II is a functional indicator of vitamin K status. In our retrospective study, all patients had increased levels in the absence of vitamin K therapy, consistent with vitamin K deficiency. Vitamin K supplementation orally decreased PIVKA-II levels by 38% but did not normalize them in most patients. The three patients with a rise in PIVKA-II levels after supplementation orally also had a decrease in FV and an increase in PT. This could be explained by further progression of the disease, since the time interval between both measurements varied from 6 to 10 years.

In the prospective study, we aimed to improve one of the causes of coagulopathy in ZSD patients, i.e., vitamin K status, by giving vitamin K IV. This had no effect on global coagulation parameters but did lower PIVKA-II levels by another 23%. This decrease was most apparent in patients with the highest levels before IV therapy. In one patient, PIVKA-II increased from 27 to 36 mAU/ml but remained within the normal range of 21–56 mAU/ml. Furthermore, thrombin generation was significantly improved, although it remains to be determined whether regular vitamin K supplementation IV will diminish bleeding complications in ZSD patients. Nevertheless, in clinical situations, including during bleeding episodes or before dental or surgical procedures, it seems to have an additional positive effect on coagulation. As ZSD patients have a high risk of bleeding, consultation with a hematologist before a surgical procedure or after trauma is important to discuss patient coagulation status and management. There is insufficient evidence, however, to give vitamin K IV on a regular basis, since it is a real burden for those children. However, it may be useful to measure PIVKA-II more often, as supportive care with vitamin K therapy orally could be improved or in some cases boosted with IV administration. More comprehensive research is indicated in larger populations to examine the clinical relevance of our laboratory findings.

In conclusion, both liver disease and vitamin K deficiency contribute to coagulopathy in ZSD patients, which particularly manifests as bleeding complications. Vitamin K deficiency is partly corrected by administering vitamin K therapy orally, and added IV administration improves vitamin K status, as shown by further decrease of PIVKA-II and improved thrombin generation.

## Abbreviations

AMC: Academic Medical Center
ALT: Alanine aminotransferase
AST: Aspartate aminotransferase
APTT: Activated partial thromboplastin time
CAT: Calibrated automated thrombography
DHCA: 3α,7α-dihydroxycholestanoic acid
FV: Factor V
FVII: Factor VII
FIX: Factor IX
FX: Factor X
IV: Intravenous
THCA: 3α,7α,12α-trihydroxycholestanoic acid
PEX: Peroxisome assembly protein
PIVKA -II: Proteins induced by vitamin K absence
PT: Prothrombin time
ZSD: Zellweger spectrum disorder
